# Brachydactyly

**DOI:** 10.1186/1750-1172-3-15

**Published:** 2008-06-13

**Authors:** Samia A Temtamy, Mona S Aglan

**Affiliations:** 1 Department of Clinical Genetics, Human Genetics and Genome Research Division, National Research Centre (NRC), El-Buhouth St., Dokki, 12311, Cairo, Egypt

## Abstract

Brachydactyly ("short digits") is a general term that refers to disproportionately short fingers and toes, and forms part of the group of limb malformations characterized by bone dysostosis. The various types of isolated brachydactyly are rare, except for types A3 and D. Brachydactyly can occur either as an isolated malformation or as a part of a complex malformation syndrome. To date, many different forms of brachydactyly have been identified. Some forms also result in short stature. In isolated brachydactyly, subtle changes elsewhere may be present. Brachydactyly may also be accompanied by other hand malformations, such as syndactyly, polydactyly, reduction defects, or symphalangism.

For the majority of isolated brachydactylies and some syndromic forms of brachydactyly, the causative gene defect has been identified. In isolated brachydactyly, the inheritance is mostly autosomal dominant with variable expressivity and penetrtance.

Diagnosis is clinical, anthropometric and radiological. Prenatal diagnosis is usually not indicated for isolated forms of brachydactyly, but may be appropriate in syndromic forms. Molecular studies of chorionic villus samples at 11 weeks of gestation and by amniocentesis after the 14^th ^week of gestation can provide antenatal diagnosis if the causative mutation in the family is known. The nature of genetic counseling depends both on the pattern of inheritance of the type of brachydactyly present in the family and on the presence or absence of accompanying symptoms.

There is no specific management or treatment that is applicable to all forms of brachydactyly. Plastic surgery is only indicated if the brachydactyly affects hand function or for cosmetic reasons, but is typically not needed. Physical therapy and ergotherapy may ameliorate hand function. Prognosis for the brachydactylies is strongly dependent on the nature of the brachydactyly, and may vary from excellent to severely influencing hand function. If brachydactyly forms part of a syndromic entity, prognosis often depends on the nature of the associated anomalies.

## Background

### Definition

The term brachydactyly is derived from the ancient Greek (brachy-: short; dactylos: digit). It indicates shortening of digits due to abnormal development of phalanges, metacarpals, or both. Brachydactyly is one of the ten categories of hand malformations classified by Temtamy & McKusick [1] in their original work on the genetics of hand malformations. In the latest International nosology and classification of genetic skeletal dysplasias, brachydactyly was included as one of the dysostosis groups affecting the limbs [2]. Dysostoses refer to abnormalities of individual bones, either in isolation or in combination with various abnormally formed bones. They are usually static and arise during blastogenesis (1^st ^8 weeks of embryonic life) [3], thus they differ from osteochondrodysplasias that usually present at a later stage of development, typically affect the skeleton in general, and may continue to evolve as a result of continuous gene functioning throughout life [3].

### Epidemiological data

The various types of isolated brachydactyly are rare, except for types A3 and D, which are common, prevalence being around 2% [1].

A search of Online Mendelian Inheritance in Man [4] using brachydactyly as key term provided 232 entries (isolated forms; syndromes with brachydactyly; skeletal dysplasias). A similar search using brachydactyly as search term in the London Medical Databases (LMD) [5] yielded 386 entities. The incidence and prevalence of these entries varied considerably.

### Classification

As in all congenital anomalies, brachydactyly can occur either as an isolated malformation or as a part of a complex malformation syndrome. In isolated brachydactyly (Table 1), subtle changes elsewhere may still be present. Brachydactyly may also go along with other hand malformations, such as syndactyly, polydactyly, reduction defects, or symphalangism.

**Table 1:** Types of isolated brachydactyly

Name	Synonyms	OMIM
**Brachydactyly type A**		
Brachydactyly type A1 (BDA1)	Farabee type brachydactyly	112500
Brachydactyly type A2 (BDA2)	Mohr-Wriedt type brachydactyly	112600
Brachydactyly type A3 (BDA3)	Brachymesophalangy V, Brachydactyly-Clinodactyly	112700
Brachydactyly type A4 (BDA4)	Brachymesophalangy II and V, Temtamy type brachydactyly	112800
Brachydactyly type A5 (BDA5)	Absent middle phalanges of digits 2–5 with nail dysplasia	112900
**Brachydactyly type B (BDB)**		113000
**Brachydactyly type C (BDC)**	Brachydactyly with hyperphalangism, Haws type brachydactyly	113100
**Brachydactyly type D (BDD)**	Stub thumb	113200
**Brachydactyly type E (BDE)**		113300
**Brachymetatarsus IV**	Metatarsus IV, short, Toes, fourth, short	113475
**Sugarman brachydactyly**	Brachydactyly with major proximal phalangeal shortening	272150
**Kirner deformity**	Dystelephalangy	128000

One of the most commonly used classifications of brachydactyly based on anatomic grounds was provided by Bell [6] and further elaborated by Temtamy & McKusick [1]. This system of classification has the advantage of being malleable to accommodate addition of any other recently discovered forms of brachydactyly mentioned in the literature. No other classifications of brachydactyly, apart from a few appearing in the Russian and Romanian literature, are known to us. Therefore, we follow here the definitions of the various types of brachydactyly as described by Temtamy and McKusick [1]. We will focus on isolated brachydactyly, although selected syndromic forms will also be discussed.

### General considerations of brachydactylies

#### • Antenatal diagnosis and genetic counseling

Prenatal diagnosis is usually not indicated for isolated forms of brachydactyly, but may be appropriate in syndromic forms. Short phalanges may not be clearly evident by fetal ultrasonography early in development, and will only be visible later on, if expressed. Molecular studies of chorionic villus sampling at 11 weeks of gestation and by amniocentesis after the 14^th ^week of gestation can provide antenatal diagnosis if the causative mutation in the family is known.

#### • Genetic counseling

The nature of genetic counseling depends both on the pattern of inheritance of the type of brachydactyly present in the family and on the presence or absence of accompanying symptoms.

If the brachydactyly follows an autosomal dominant pattern of inheritance, the chance of recurrence in offspring of affected individuals is 50% regardless of sex. Variability in severity and incomplete penetrance are common in several types of brachydactyly.

If the brachydactyly follows an autosomal recessive pattern of inheritance, the risk of recurrence in the sib of an affected case is 25%. Although variable symptoms may still be present in affected sibs, variability is usually smaller in autosomal recessive forms.

#### • Management including treatment

There is no general, specific management or treatment that is applicable to all forms of brachydactyly. Plastic surgery is only indicated if the brachydactyly affects hand function or for cosmetic reasons, but is typically not needed. Physical therapy and ergotherapy may ameliorate hand function.

#### • Prognosis

Prognosis for the brachydactylies is strongly dependent on the nature of the brachydactyly, and may vary from excellent to severely influencing hand function. If brachydactyly forms part of a syndromic entity, prognosis often depends on the nature of the associated anomalies.

## Types of isolated brachydactyly

**Figure 1:**
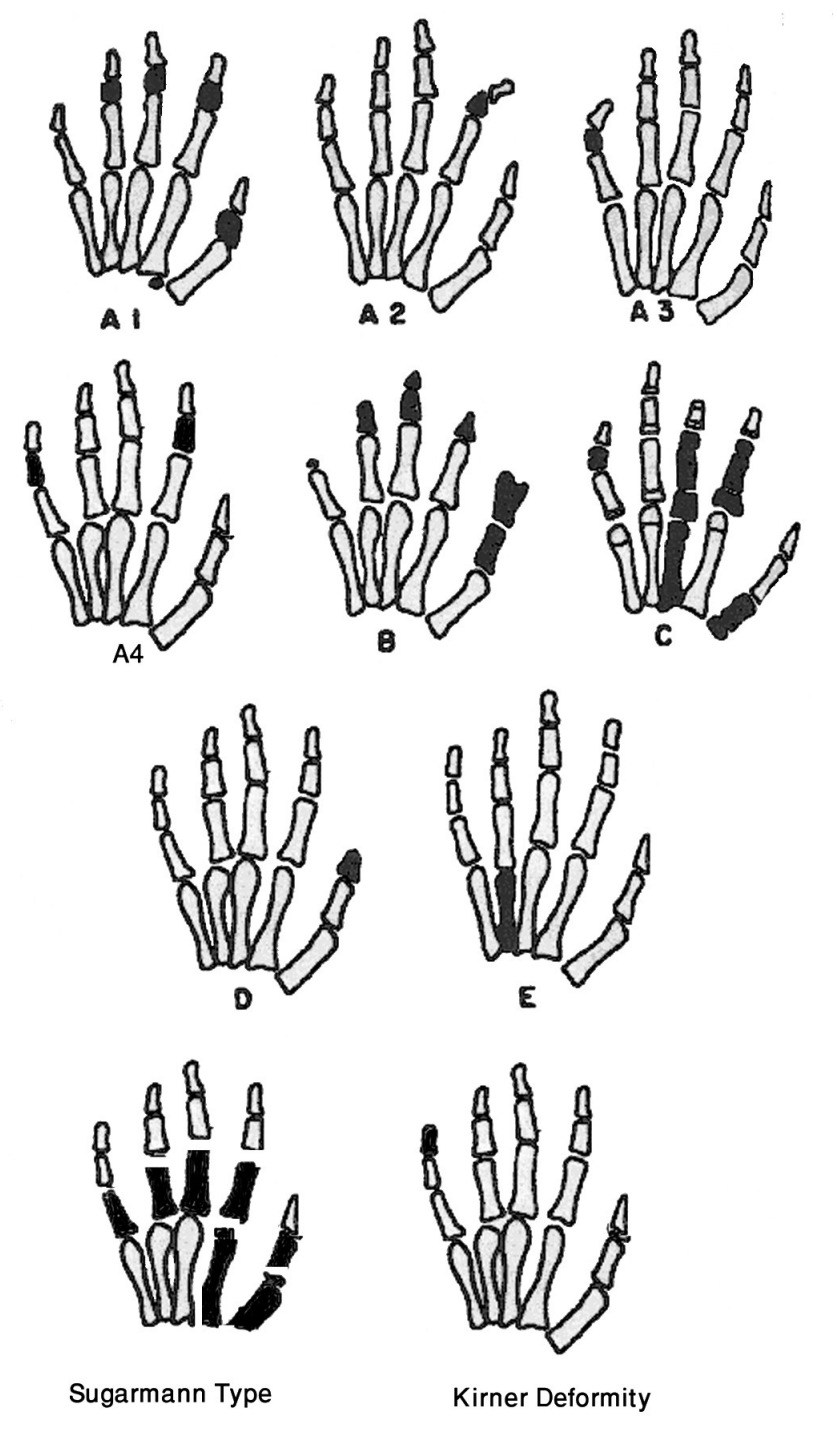
**A diagram showing main types of isolated brachydactyly**. Modified from Temtamy & McKusick [1].

Figure 1 illustrates the changes in the main types of isolated brachydactyly. Table 2 shows the genomics of isolated brachydactyly.

**Table 2:** Genomics of isolated brachydactyly

**Type**	**OMIM**	**Gene Name**	**Gene Locus**	**Reference**
BDA1	112500	*IHH BDA1B*	2q35-q35 5p13.3-p13.2	Gao *et al*. [12] Kirkpatrick *et al*. [14]
BDA2	112600	*BMPR1B GDF5*	4q21-q20 20q11.2	Lehmann *et al*. [17] Kjaer *et al*. [18]
BDA4	112800	*HOXD13*	2q31-q32	Zhao *et al*. [26]
BDB	113000	*ROR2 NOG*	9q22 4q23-q24	Schwabe *et al*. [30] Lehmann *et al*. [31]
BDC	113100	*CDMP1*	20q11.2	Polinkovsky *et al*. [37]
BDD	113200	*HOXD13*	2q31-q32	Johnson *et al*. [50]
BDE	113300	*HOXD13*	2q31-q32	Johnson *et al*. [50]

### 1. Brachydactyly type A (BDA)

shortening is confined to middle phalanges. Depending on the affected digits, BDA is subdivided into:

#### • Brachydactyly type A1 (BDA1)

(OMIM: 112500) (syn: *FARABEE TYPE BRACHYDACTYLY*)

##### Clinical description

Early in 1900's, Farabee and Drinkwater described a number of families with BDA1. In BDA1, middle phalanges of all digits are variably short or rudimentary and are occasionally fused with terminal phalanges. The proximal phalanges of the thumbs and big toes are short.

According to the degree of shortening, Drinkwater [7-9] identified two varieties, severe and minor brachydactyly. Many reports described families in which both varieties occurred in various affected members. In the severe variety, the fingers are about half of the normal length, the middle phalanges of all digits are either absent or very hypoplastic and might be fused with terminal phalanges (terminal symphalangism). In the mild variety of brachydactyly, the hypoplasia of the middle phalanges is less severe with more affection of the index and little fingers. Distal symphalangism, when present, is confined to the little finger. In both varieties of brachydactyly, the affected individuals are significantly shorter than their normal sibs. Armour *et al*. [10] reported a family with mild brachydactyly type A1 that, except for short stature, was not associated with additional clinical features. The authors illustrated the usefulness of the metacarpophalangeal profiles.

##### Epidemiology

No epidemiological studies have been reported. It is a rare hand malformation with only few pedigrees reported in the literature [4].

##### Diagnosis and diagnostic methods

Type A1 brachydactyly can be diagnosed by clinical, anthropometric and radiologic evaluation of both hands. X-ray of hands on postero-anterior (PA) view show the selective distribution of the hypoplasia and aplasia of the middle phalanges. Molecular studies are helpful (see below).

##### Inheritance

**Figure 2:**
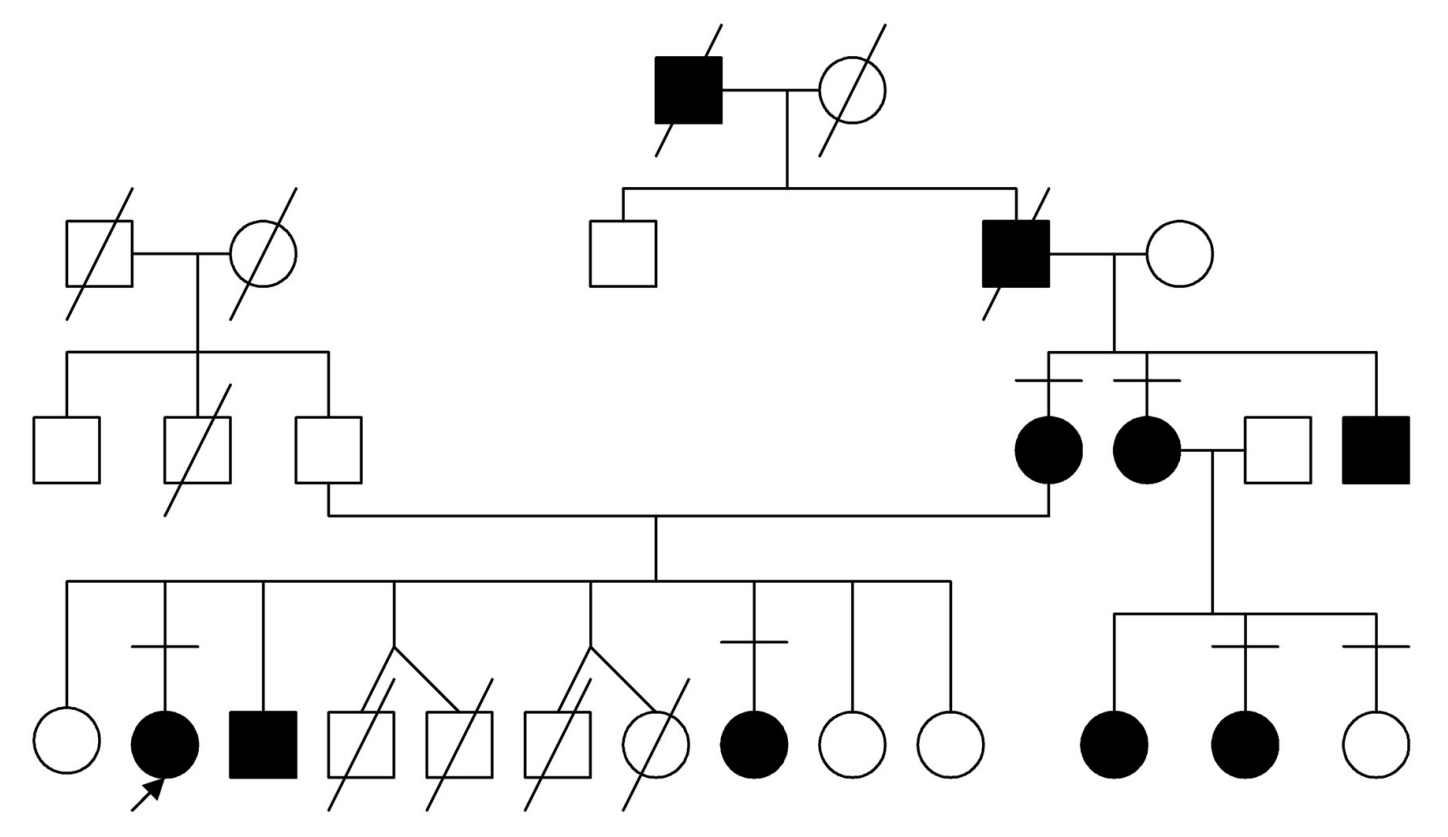
Pedigree of an Egyptian family with BDA1 denoting autosomal dominant inheritance. (Limb Malformations & Skeletal Dysplasia Clinic, Medical Services Unit (MSU), NRC).

This type of hand deformity was the first human trait interpreted in terms of Mendelian dominant inheritance [11]. Figure 2 illustrates the autosomal dominant inheritance in an Egyptian family with type A1 brachydactyly.

##### Molecular genetics

Type A1 brachydactyly can be caused by mutations in the Indian hedgehog gene (*IHH*) located on chromosome 2q35-36 [12]. This supports the hypothesis that *IHH *plays a pivotal role in normal human skeletogenesis [13]. Another locus for this phenotype, designated BDA1B has been identified on chromosome 5p13.3-p13.2 [14].

#### • Brachydactyly type A2 (BDA2)

(OMIM: 112600) (syn: *MOHR-WRIEDT TYPE BRACHYDACTYLY*)

##### Clinical description

BDA2 is characterized by hypoplasia/aplasia of the 2^nd ^middle phalanx of the index finger and sometimes little finger. It was first described by Mohr and Wriedt [15]. Characteristically, affected individuals have a triangular-shaped middle phalanx in the index fingers and second toes. In severely affected cases, the index finger is curved radially. Deformity of the 2^nd ^toe is a more consistent finding than deformity of the index finger.

The big toes show malformation of the proximal phalanx resulting in fibular deviation of the distal phalanx, while all other toes have rudimentary middle phalanges causing tibial deflection of their distal phalanges.

Jones [16] suggested the designation "delta phalanx" for the triangular middle phalanx. On surgical exploration, he found that the epiphysis of the triangular phalanx was continuous, running from the proximal to the distal end of the phalanx along the shortened side. Thus, growth can occur only outward leading to persistent angulations and little gain in length. Temtamy and McKusick [1] reported a family, the fourth in the literature, with 16 affected members in four generations. One member of the family had severe bilateral digital anomalies and may have represented homozygosity for the mutant gene because his father, who had the usual phenotype of A2 brachydactyly, was married to a normal relative.

##### Epidemiology

A very rare digital malformation. No epidemiologic studies available. Reported pedigrees are summarized in OMIM [4].

##### Diagnosis and diagnostic methods

Clinical evaluation of the hands and curved index finger, together with the second toe, also seen in X-rays. This is seen as triangular shaped middle phalanx of the index fingers and second toes.

##### Inheritance

Studied families demonstrate autosomal dominant inheritance.

##### Molecular genetics

Type A2 brachydactyly can be caused by mutation in the human bone morphogenetic protein receptor 1B gene (*BMPR1B*) on chromosome 4q, that affect cartilage formation in a dominant-negative manner [17]. Kjaer *et al*. [18] found that sparing of the 4^th ^finger distinguishes the Mohr-Wriedt type BDA2 from BDA2 caused by mutations in *BMPR1B *and concluded that the growth and differentiation factor 5 gene (*GDF5*) on chromosome 20q11 is a novel BDA2 causing gene. Mutations in *GDF5 *alter the receptor binding affinities and can also cause symphalangism [19].

#### • Brachydactyly type A3 (BDA3)

(OMIM: 112700) (syn: *BRACHYMESOPHALANGY V, BRACHYDACTYLY-CLINODACTYLY*)

##### Clinical description

BDA3 is characterized by shortening of the middle phalanx of the little finger. Slanting of the distal articular surface of the middle phalanx leads to radial deflection of the distal phalanx. It is not always associated with clinodactyly. A single flexion crease of the little finger indicates a short or absent middle phalanx. This type should be differentiated from other types of crooked little fingers, namely Kirner deformity and camptodactyly, in the former there is radial bowing of the terminal phalanx due to curving of its shaft. Camptodactyly is a flexure contracture deformity of the interphalangeal joints.

##### Epidemiology

Type A3 brachydactyly is very common. Sugiura *et al*. [20] found a frequency of 21% among Japanese school children. The frequency varied in different populations between 3.4% and 21% [1].

##### Diagnosis and diagnostic methods

Hertzog [21] defined it as middle phalanx V less than half the length of middle phalanx IV. Garn *et al*. [22] provided percentiles for the length of the phalanges and metacarpals for various age groups making the diagnosis of brachydactyly possible in doubtful cases. Clinodactyly is not always associated with a short middle phalanx and vice-versa. A single flexion crease of the little finger indicates a short or absent middle phalanx. Other associated anomalies should be sought to diagnose syndromic associations [1].

##### Inheritance

This type is autosomal dominant with reduced penetrance.

##### Molecular genetics

no gene or locus for BDA3 has yet been identified.

#### • Brachydactyly type A4 (BDA4)

(OMIM: 112800) (syn: *BRACHYMESOPHALANGY II AND V, TEMTAMY TYPE BRACHYDACTYLY*)

##### Clinical description

Temtamy and McKusick [1] studied a pedigree with an unusual type of brachydactyly in four generations. The main features were brachymesophalangy affecting mainly the 2^nd ^and 5^th ^digits. When the 4^th ^digit was affected, it showed an abnormally shaped middle phalanx leading to radial deviation of the distal phalanx. The feet also showed absence of middle phalanges of the lateral four toes. The propositus had congenital talipes calcaneovalgus. One of Bell's unclassified pedigrees representing the same type of brachydactyly was reported by Jeanselme *et al*. [23] with affected members in four generations. The affected members had brachydactyly of the 2^nd ^and 5^th ^fingers due to brachymesophalangy, and one affected member had club foot. Stiles and Schalck [24] described a family in which many members of four generations had ulnar curvature of the second finger. Usually the 5^th ^finger, and sometimes also the 4^th^, showed at least mild radial curvature. Ohzeki *et al*. [25] reported this form of brachydactyly in a Japanese mother and daughter who were also short of stature.

##### Epidemiology

This type is apparently rare. Only few pedigrees were reported [4].

##### Diagnosis and diagnostic methods

Clinical evaluation of digits. Radiological examination and pattern profile analysis for the length of the phalanges.

##### Molecular genetics

Mutations in the homeobox containing gene (*HOXD13*) can give rise to limb malformations with variable expressivity and a wide spectrum of clinical manifestations including synpolydactyly and brachydactyies types D and E. Zhao *et al*. [26] found a link between *HOXD13 *and two additional limb phenotypes – syndactyly type V and brachydactyly type A4, and suggested the term "HOXD13 limb morphopathies" for the spectrum of limb disorders caused by *HOXD13 *mutations.

#### • Brachydactyly type A5 (BDA5)

(OMIM: 112900) (syn: *ABSENT MIDDLE PHALANGES OF DIGITS 2–5 WITH NAIL DYSPLASIA*)

Absence of the middle phalanges and nail dysplasia with duplicated terminal phalanx of the thumb was reported by Bass [27] and Cuevas-Sosa and Garcia-Segur [28]. We think that this type can be included in type B brachydactyly.

### 2. Brachydactyly type B (BDB)

(OMIM: 113000)

#### Clinical description

There is absence or hypoplasia of the terminal parts of the index to little fingers with complete absence of fingernails. The thumbs are always intact but frequently show flattening, splitting or duplication of the distal phalanges. Digits are less severely affected on the radial side of the hand compared to those on the ulnar side. The feet are similarly but less severely affected. The deformity is symmetric. There is soft tissue syndactyly, symphalangism, carpal and/or tarsal fusions and shortening of metacarpals and/or metatarsals.

#### Epidemiology

This is a rare hand malformation with only a few published pedigrees in the world literature [4].

#### Diagnosis and diagnostic methods

Clinical examination of the thumb anomalies and distal phalangeal hypoplasia, and absence of nails sometimes with associated distal symphalangism. Radiological examination of the hands and pattern profile analysis.

#### Inheritance

MacKinder [29] described this hand deformity in six generations of a family. All studied cases in the literature demonstrated an autosomal dominant pattern of inheritance with variable severity. The range of variability of the trait is less extreme among various affected members of the same pedigree. Temtamy and McKusick [1] reported a pedigree with 11 affected individuals in 6 generations, also with autosomal dominant inheritance and variable expressivity.

#### Molecular genetics

In the majority of cases, BDB is caused by heterozygous truncating mutations in the receptor kinase-like orphan receptor 2 gene (*ROR2*) on 9q22. Patients affected with the distal mutations have a more severe phenotype than those with the proximal mutations [30]. In a subset of *ROR2*-negative patients with BDB and additional occurrence of proximal symphalangism and carpal synostosis, Lehmann *et al*. [31] identified different mutations in the bone morphogenetic protein (*BMP*) antagonist NOGGIN (*NOG*). The authors argued for a functional connection between *BMP *and *ROR2 *signaling and support the findings of a modulating effect of *ROR2 *on the *BMP*-receptor pathway through the formation of a heteromeric complex of the receptors at the cell surface. Autosomal recessive Robinow syndrome is also caused by mutations in the *ROR2 *gene. The BDB phenotype, as well as the location and nature of the BDB mutations, suggest a specific mutational effect that can not be explained by simple haploinsufficiency and that is distinct from that in Robinow syndrome.

### 3. Brachydactyly type C (BDC)

(OMIM: 113100) (syn: *BRACHYDACTYLY WITH HYPERPHALANGISM, HAWS TYPE*)

#### Clinical description

The hand deformity is characterized by brachymesophalangy of the index, middle and little fingers with hyperphalangy of the index and middle finger and shortening of the 1^st ^metacarpal. The ring finger is usually the longest digit. The proximal phalanx of the index finger has an anomalous configuration resulting in its ulnar deflection. Short metacarpals and symphalangism are occasionally present. The feet are either normal or show ordinary brachydactyly. Temtamy and McKusick [1] reported one family with 10 affected members from 3 generations representing autosomal dominant inheritance and variable expressivity. The proband and her identical twin were similarly affected, with mild variability. Considerable intra- and interfamilial variation has been observed in type C brachydactyly [32].

Radiographic findings suggest a disturbance in chondrification and ossification with maximal effect on the development of the epiphyses and shafts of certain bones of the digits. Schwabe *et al*. [33] found mild shortening in hand radiographs of heterozygous mutation carriers. Castriota-Scanderbeg *et al*. [34] described a woman and her daughter with BDC. The child had angle-shaped appearance of the proximal phalanges of index and middle fingers in one hand and more typical triangular epiphyses with elongation of their radial side at the opposite hand suggesting that this peculiar phalangeal configuration occurs as a transitory event in early or mid childhood in phalanges that are marked by severe ossification delay.

#### Epidemiology

This type of brachydactyly is rare with only few reported pedigrees [4].

#### Diagnosis and diagnostic methods

Clinical evaluation of hands. X-rays of hands and pattern profile analysis. The ring finger is always the longest, longer than the index (hyperphalangism detected on radiological examination).

#### Inheritance

Many studies support an autosomal dominant trait. Baraitser and Burn [35] described an affected brother and sister whose Iraqi first-cousin parents were unaffected, raising the possibility of autosomal recessive inheritance of this phenotype. Schwabe *et al*. [33] identified a mutation in the *GDF5 *gene in a consanguineous Turkish kindred with BDC. Homozygous offspring exhibited brachymesophalangy and hyperphalangy of the second, third, and fifth fingers with some phenotypic variability while all heterozygous mutation carriers showed mild shortening of metacarpals 4 and 5, suggesting a semidominant pattern of inheritance. Intra-familial variability and the observation of skipped generations in some families indicate that BDC may not be a true autosomal dominant condition due to mutation of a single gene [36].

#### Molecular genetics

Heterozygous mutations of cartilage derived morphogenetic protein 1 (*CDMP1*), also known as growth/differentiation factor-5 gene (*GDF5*), resulting in loss of function have been reported in BDC [37]. Galjaard *et al*. [32] suggested that factors other than locus heterogeneity, such as genetic modifiers and/or environmental factors must play a role in phenotypic variability. Everman *et al*. [38] described heterozygous mutations in additional families with BDC and revealed non-penetrance in a mutation carrier. Schwabe *et al*. [33] described a novel missense mutation in the prodomain of *CDMP1 *indicating an important function of the prodomain for the folding, secretion and availability of biologically active *CDMP1*.

*There is a considerable degree of phenotypic overlap between BDA2, BDB and BDC. Mutations in the BMPR1B gene cause BDA2, mutations in the ROR2 gene cause BDB and mutations in the GDF5 gene cause BDC. Sammar et al*. [39]*demonstrated that all three components are involved in GDF5-induced regulation of chondrogenesis. They found that the functional interaction of ROR2 with GDF5 and BMPR1B is genetically confirmed by the presence of epistatic effects in crosses of ROR2, BMPR1B and GDF5 deficient mice. Lehmann et al*. [40]*demonstrated that disturbances of the NOG-GDF5-BMPR1B signaling cascade can result in either a BDC/symphalangism like phenotype or BDA2 depending on the quantitative effect and mode of action of the specific mutations within the same functional pathway*.

### 4. Brachydactyly type D (BDD)

(OMIM: 113200) (syn: *STUB THUMB*)

#### Clinical description

Characteristically, the distal phalanx of the thumb alone is shortened. There are various degrees of thumb shortening, either unilaterally or bilaterally. It has been noted that the base of the distal phalanx is broader than the surface of the proximal phalanx to which it articulates, and that the distal end of the bone often shows some hyperplasia. Other investigations confirmed that the short distal phalanx of the thumb results from early closure of its epiphyses.

#### Epidemiology

This type of brachydactyly is common. The prevalence varies in different populations, ranging from 0.41% to 4.0%. Particularly high prevalence was reported among Israeli Arabs and in the Japanese population [1].

#### Diagnosis and diagnostic methods

Clinical evaluation and radiographs show a broad and short distal phalanx of thumbs, unilateral or bilateral. The distal phalanges of other digits may also be broad and short. Great toes may be similarly affected.

#### Inheritance

Breitenbecher [41] described the trait as autosomal dominant. Various other studies supported an autosomal dominant pattern with reduced penetrance.

#### Molecular genetics

no gene or locus for BDD has yet been identified.

### 5. Brachydactyly type E (BDE)

(OMIM: 113300)

#### Clinical description

**Figure 3:**
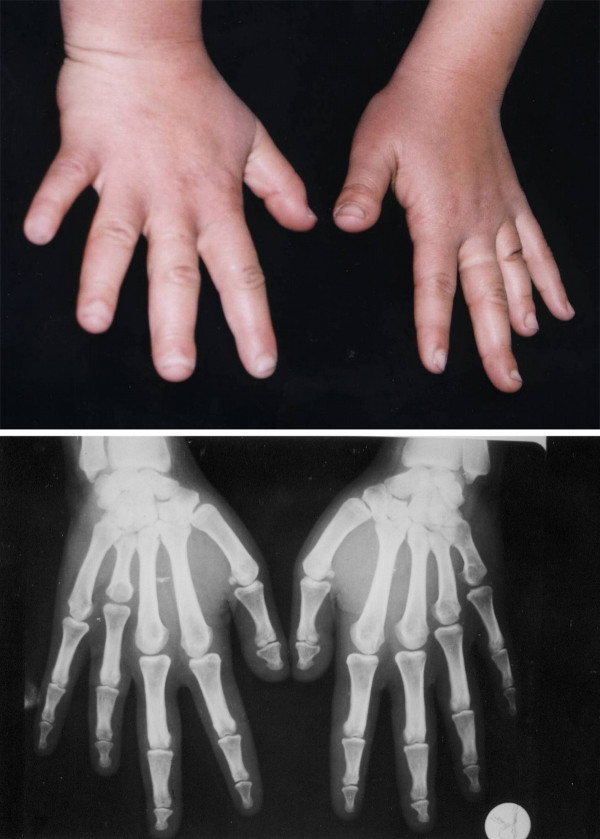
**Hands and X-ray hands of an Egyptian patient with BDE**. Note the short 4th and 5th metacarpals of hands. (Limb Malformations & Skeletal Dysplasia Clinic, MSU, NRC).

Variable shortening of the metacarpals with more or less normal length of phalanges (Fig. 3). Occasionally, the metatarsals are also short. This results from hypoplastic and partially fused metacarpal epiphyses, visible on radiographs. The terminal phalanges are often short. Hyperextensibility of the hand joints is frequently a striking feature. The axial triradius is often relatively high. Affected individuals are slightly short of stature. Temtamy and McKusick [1] reported a family with 17 affected members in three generations confirming autosomal dominant inheritance and variable expressivity.

Short metacarpals and metatarsals are frequent findings in other types of brachydactyly and are features of several genetic syndromes. Some individuals with BDE are moderately short of stature and have round facies but do not have ectopic calcification, mental retardation or cataract as in pseudopseudohypoparathyroidism (PPHP) which is otherwise a clinically similar entity. Poznanski *et al*. [42] concluded that 'brachydactyly E is indistinguishable radiologically from the PHP-PPHP syndrome'. Short stature, round facies, secundum type of atrial septal defect and BDE (in which shortening of the metacarpals was most pronounced but not limited to the 4th metacarpal), were described in a kindred by Czeizel and Göblyös [43].

Hertzog [44] suggested that there are at least three subtypes of BDE: **E1**, in which shortening is limited to the fourth metacarpals and/or metatarsals [45]; **E2**, in which variable combinations of metacarpals are involved, with shortening also of the first and third distal and the second and fifth middle phalanges [46] and **E3**, a dubious category which may have a variable combination of short metacarpals without phalangeal involvement.

Pitt and Williams [47] described a 'new' type of brachydactyly in 12 members of four generations combining features of types B and E with hypoplasia of the distal phalanges of the ulnar side of the hand and shortening of one or more metacarpals. The subjects were, however, not short of stature as in type E. Male-to-male transmission was noted in several instances. The authors called this phenotype brachydactyly type Ballard after the name of the family. Jensen and Hoo [48] described a similar family but pointed out that the manifestations in both families strongly resembled type E brachydactyly and were likely to represent a variant form of this phenotype. In particular, they considered the condition in both families to be compatible with the E2 subtype, as suggested by Hertzog [44].

#### Epidemiology

Rare as an isolated anomaly [1].

#### Diagnosis and diagnostic methods

Clinical and radiological evaluation of hand bones. Short 4^th ^metacarpal can be diagnosed by a positive metacarpal sign. On clinical examination (using a pencil touching the heads of the 3^rd ^and 5^th ^metacarpals of the back of the closed fist), the 4^th ^metacarpal is receding. The same sign can be identified on X-rays by drawing a line to touch tips of the heads of the 3^rd ^and 5^th ^metacarpals (Fig. 3).

#### Inheritance

As an isolated anomaly, type E brachydactyly is inherited as an autosomal dominant trait with variable expressivity and variable involvement of the metacarpals and/or metatarsals, with frequent bilateral asymmetry. This phenotype is a useful example of genetic heterogeneity, because in addition to the autosomal dominant isolated type and Albright hereditary osteodystrophy, it also occurs with Turner syndrome.

Brachydactyly type E occurs with short stature and severe hypertension as an autosomal dominant syndrome (OMIM: 112410). Mollica *et al*. [49] reported a kindred in which six individuals had anetodermia (macular atrophy of the skin), eight had multiple exostoses, and two had type E brachydactyly seemingly unrelated to exostoses. In all, 10 members had one or more of these features. The authors suggested that this is an autosomal dominant syndrome.

#### Molecular genetics

Brachydactyly type E as an isolated abnormality has not been mapped with confidence. Its occurrence was one feature of what appeared to be a contiguous gene syndrome due to a cytogenetically visible *de novo *deletion of 2q37. Johnson *et al*. [50] described missense mutations in the homeodomain of *HOXD13 *in two cases with distinctive limb phenotypes exhibiting overlap with BDD and BDE.

### 6. Brachymetatarsus IV

(OMIM: 113475) (syn: METATARSUS IV, SHORT TOE, FOURTH, SHORT)

#### Clinical description

Ray and Haldane [51] described short fourth metatarsi resulting in unilateral or bilateral short fourth toes in 206 persons in Northeastern India with no instance of short metacarpals, distinguishing this form from BDE. The authors concluded that the trait is autosomal dominant with approximately 27% penetrance.

#### Epidemiology

Not known, probably not rare.

#### Diagnosis and diagnostic methods

Clinical and radiological evaluation of feet will show short 4^th ^metatarsals.

### 7. Sugarman brachydactyly

(OMIM: 272150) (syn: *BRACHYDACTYLY WITH MAJOR PROXIMAL PHALANGEAL SHORTENING*)

#### Clinical description

Sugarman *et al*. [52] described a new form of brachydactyly of which a conspicuous feature was a non-articulating great toe which was set dorsal and proximal to the usual position. The great toes were amputated. The fingers were very short and had no motion at the proximal interphalangeal joints. The consanguinity in the family and the presence of seven other affected members among the patient's relatives made autosomal recessive inheritance likely.

Radiographs of the case described by Sugarman *et al*. [52] were provided by Fujimoto *et al*. [53]. The hands had bilateral double first metacarpals. The fifth fingers had only two phalanges; the proximal and distal phalanges did not show bony fusion. The mother's hands were normal. Fujimoto *et al*. [53] described an infant girl, sister of Sugarman's proband (with the same mother but a different father) who showed brachydactyly with major shortening in the proximal phalanges. The first toes were proximally placed and medially curved. A paternal aunt of the mother had three children with the same anomaly. Consanguinity of the paternal aunt and her husband was claimed by Sugarman *et al*. [52], but could not be confirmed by Fujimoto *et al*. [53] who proposed autosomal dominant inheritance with reduced penetrance.

#### Epidemiology

Extremely rare. Only one affected family reported twice by both Sugarman *et al*. [52] and by Fujimoto *et al*. [53].

#### Diagnosis and diagnostic methods

By clinical and radiological changes.

### 8. Kirner deformity

(OMIM: 128000) (syn: *DYSTELEPHALANGY*)

#### Clinical description

This malformation of the little finger was first described by Kirner in 1972 [54]. It consists of radial bowing of the terminal phalanx. The tip of the little finger points towards the thenar eminence. This malformation is usually bilateral.

#### Epidemiology

In an isolated Japanese village, Saito [55] found a frequency of 0.46% in males and 0.63% in females. David and Burwood [56] estimated an incidence of 1 in 410, with a higher prevalence in females than in males.

#### Diagnosis and diagnostic methods

Clinical examination shows radial bowing of the terminal phalanx of the little finger, which is short. Radiologically, the following manifestations were listed by David and Burwood [56]:

• A well-defined radiolucent nidus of between 1 and 2 mm in diameter present in the terminal tuft.

• The diaphysis of the distal phalanx is short relative to the middle phalanx in a ventroradial angulation.

• The diaphysis is sclerotic with sparing of both the epiphysis and the tuft.

• The dorsopalmar diaphysis is reduced.

• Apparent overgrowth of the epiphysis of the distal phalanx is demonstrated at the ventral margin. They noted an anterior spur, which in the AP projection may mimic a cone shaped epiphysis. They considered the deformity of the little finger as congenital with lack of the radiological signs before epiphyseal ossification at about the age of 2 years.

#### Inheritance

Autosomal dominant with incomplete penetrance.

#### Molecular genetics

no gene or locus for Kirner deformity has yet been identified.

## Brachydactyly as part of a syndrome

**Figure 4:**
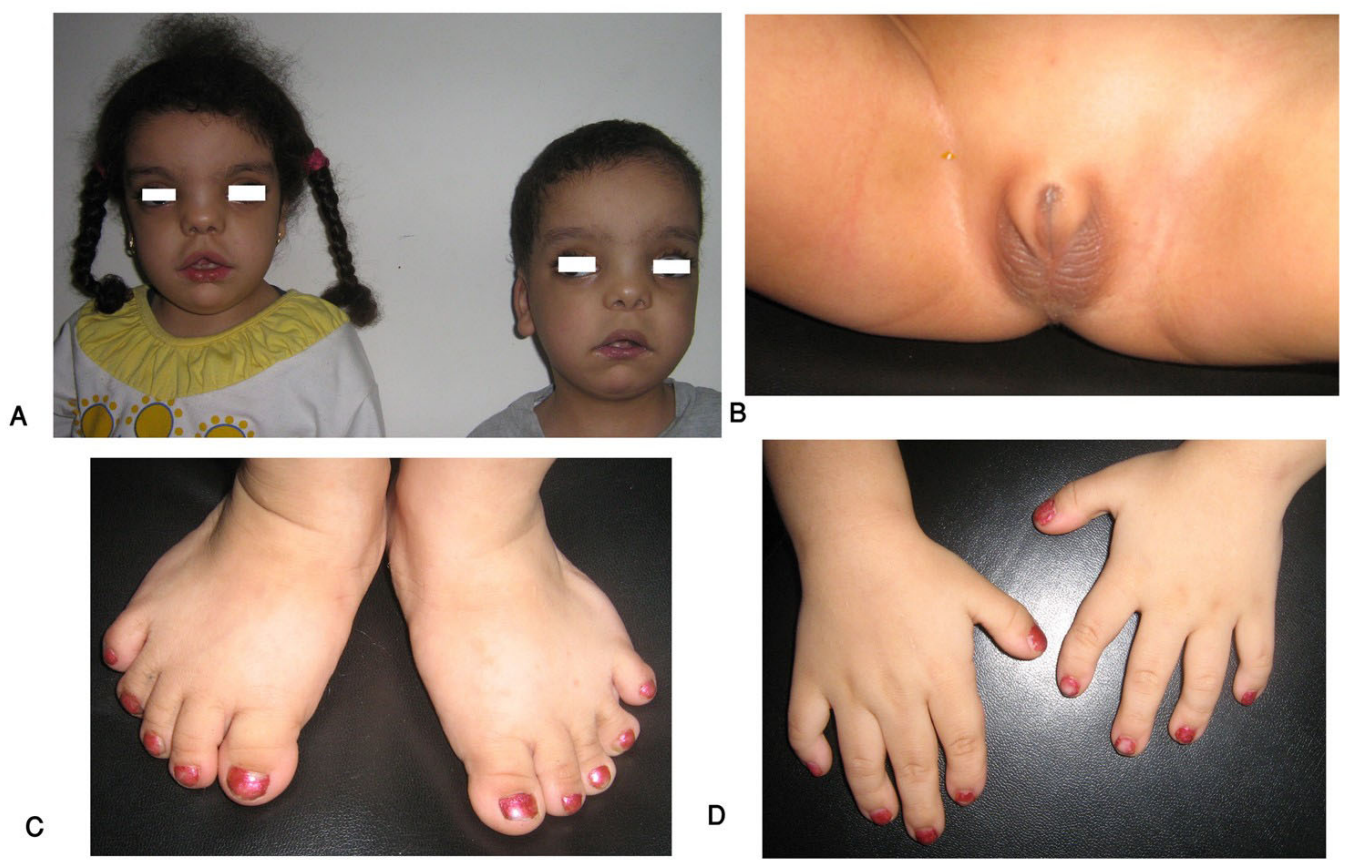
**Egyptian sibs with autosomal recessive Robinow syndrome. Note characteristic fetal face (A), hypogenitalism (B) and brachydactyly of hands and feet (C, D)**. (Limb Malformations & Skeletal Dysplasia Clinic, MSU, NRC).

**Figure 5:**
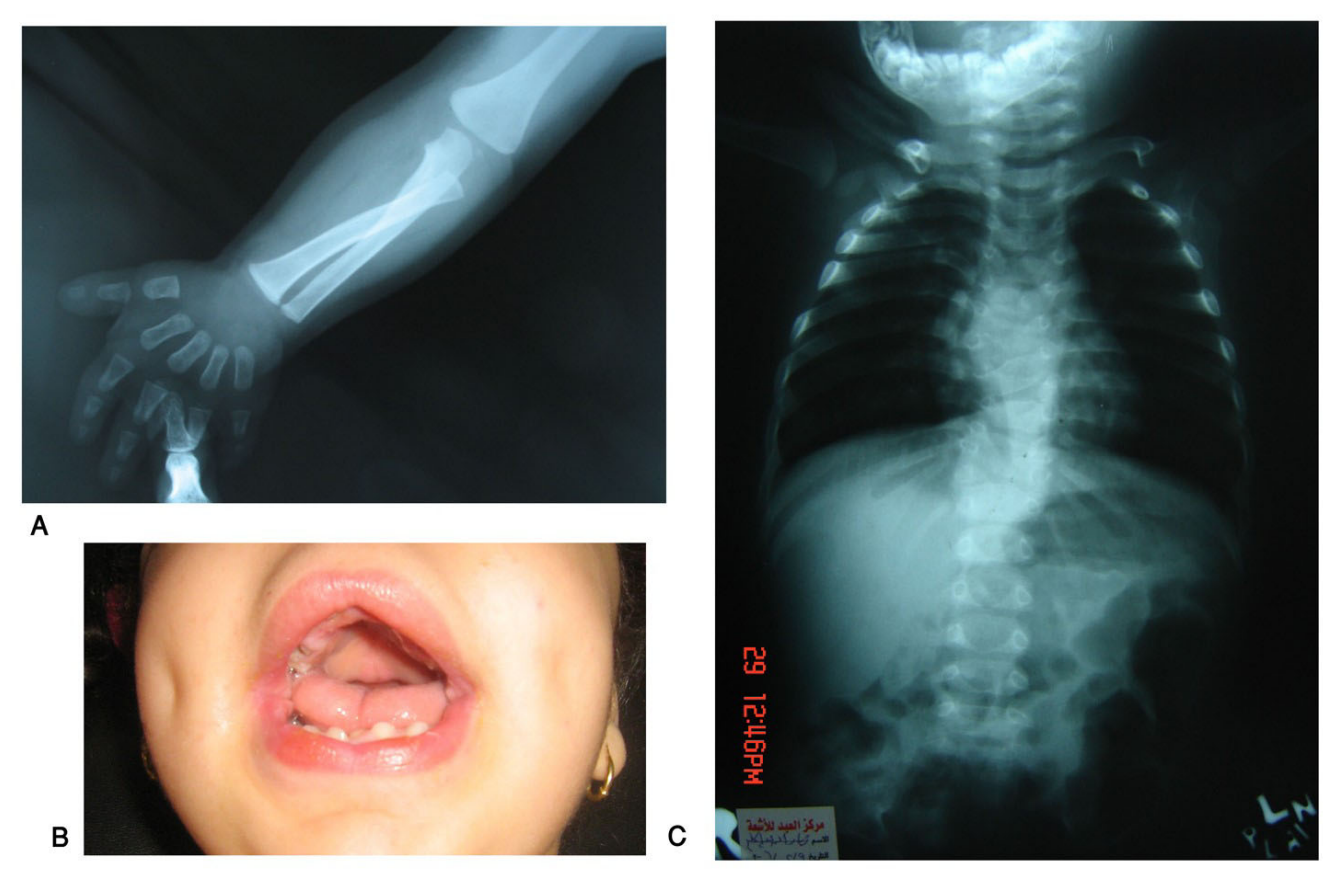
**Autosomal recessive Robinow syndrome**. X-ray upper limbs and hands showing mesomelic shortening and brachydactyly (A), gingival hyperplasia (B) and X-ray vertebrae showing hemivertebrae and vertebral fusion. (Limb Malformations & Skeletal Dysplasia Clinic, MSU, NRC).

**Figure 6:**
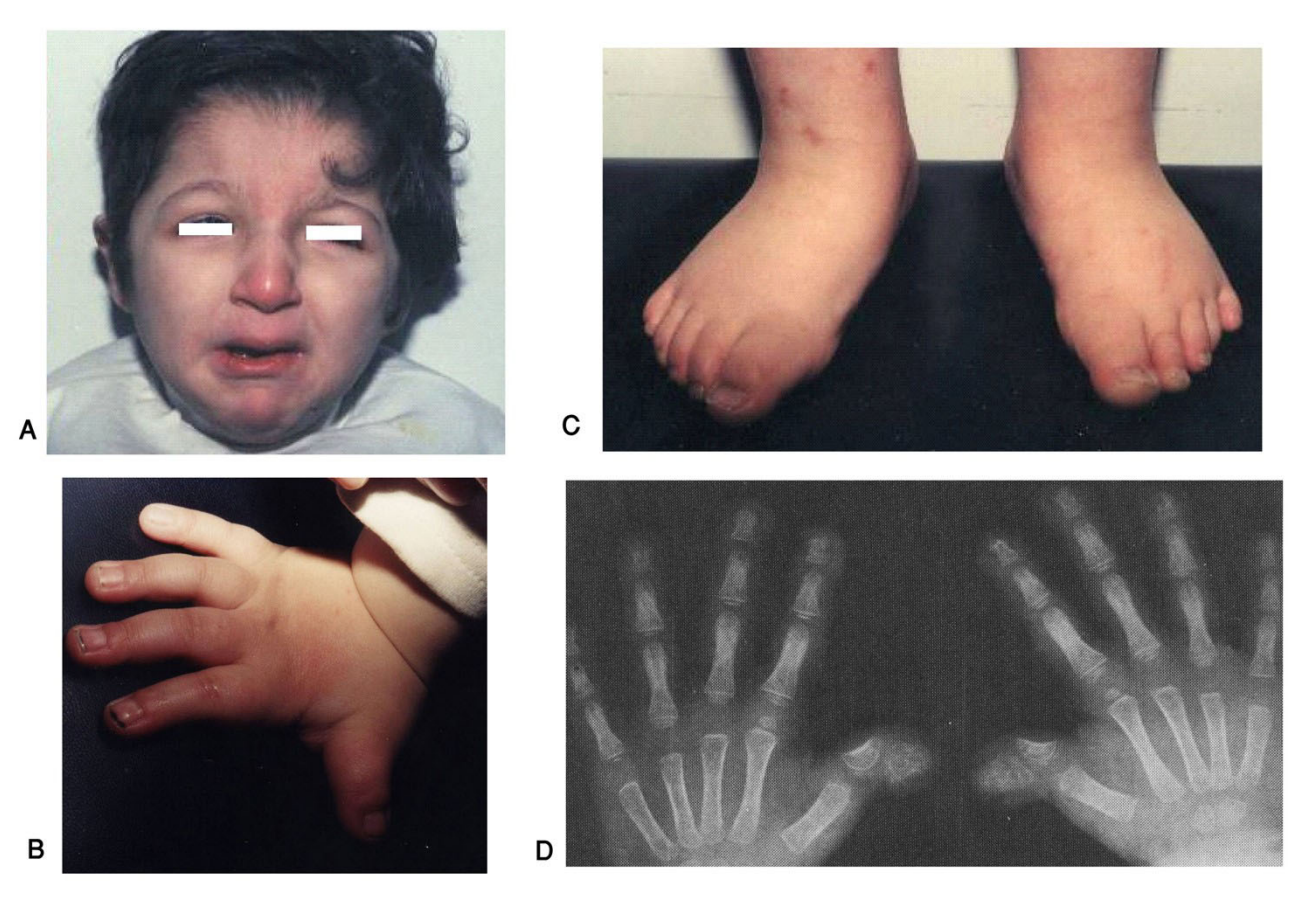
**An Egyptian patient with Rubinstein-Taybi syndrome.** Facial features (A), left hand and feet showing broad thumb and big toes (B, C) and X-ray of both hands showing short broad thumbs (D). (Limb Malformations & Skeletal Dysplasia Clinic, MSU, NRC).

**Figure 7:**
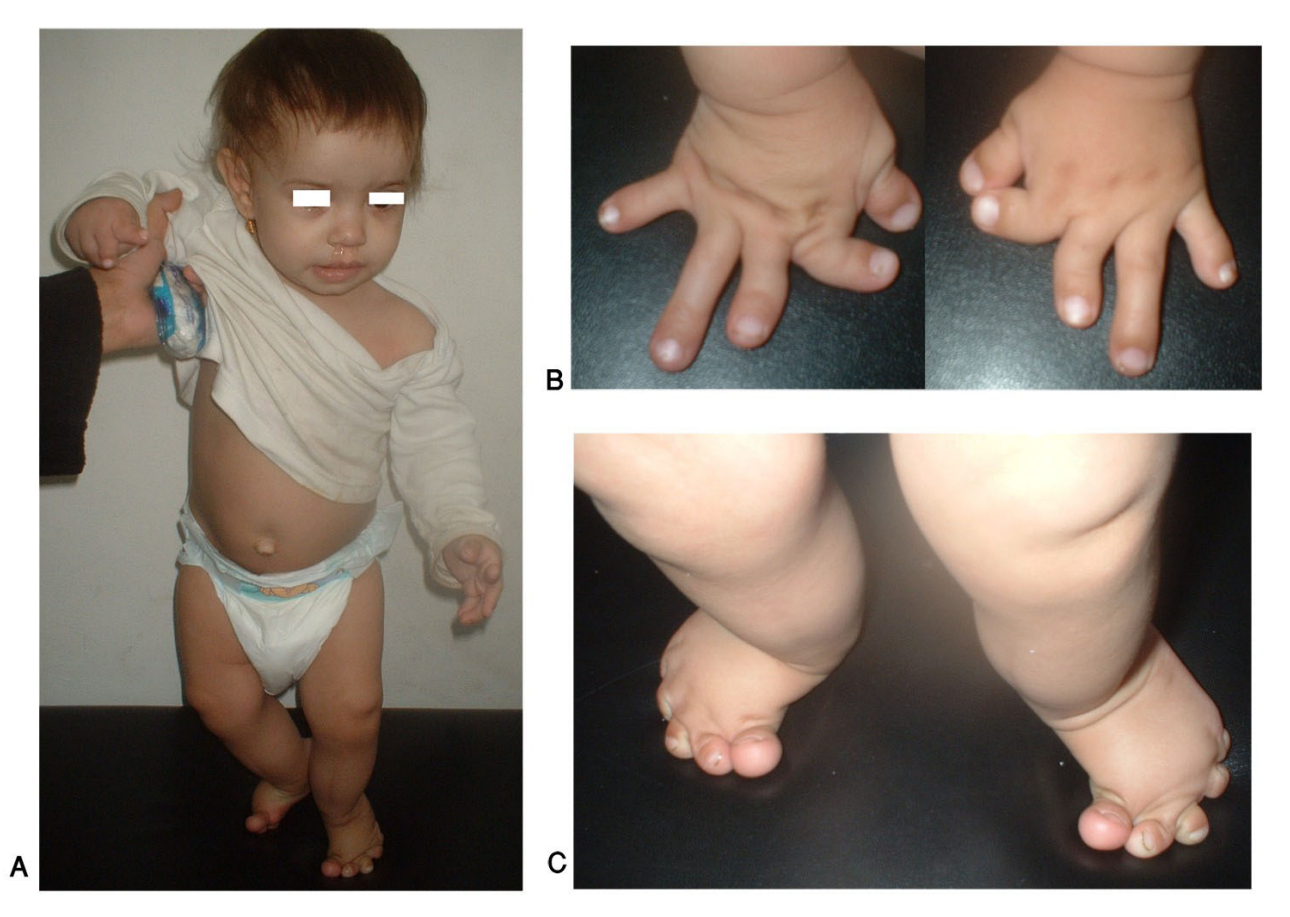
**An Egyptian patient with du Pan syndrome**. Frontal view of whole body (A), hands and feet with severe complex brachydactyly (B, C). (Limb Malformations & Skeletal Dysplasia Clinic, MSU, NRC).

**Figure 8:**
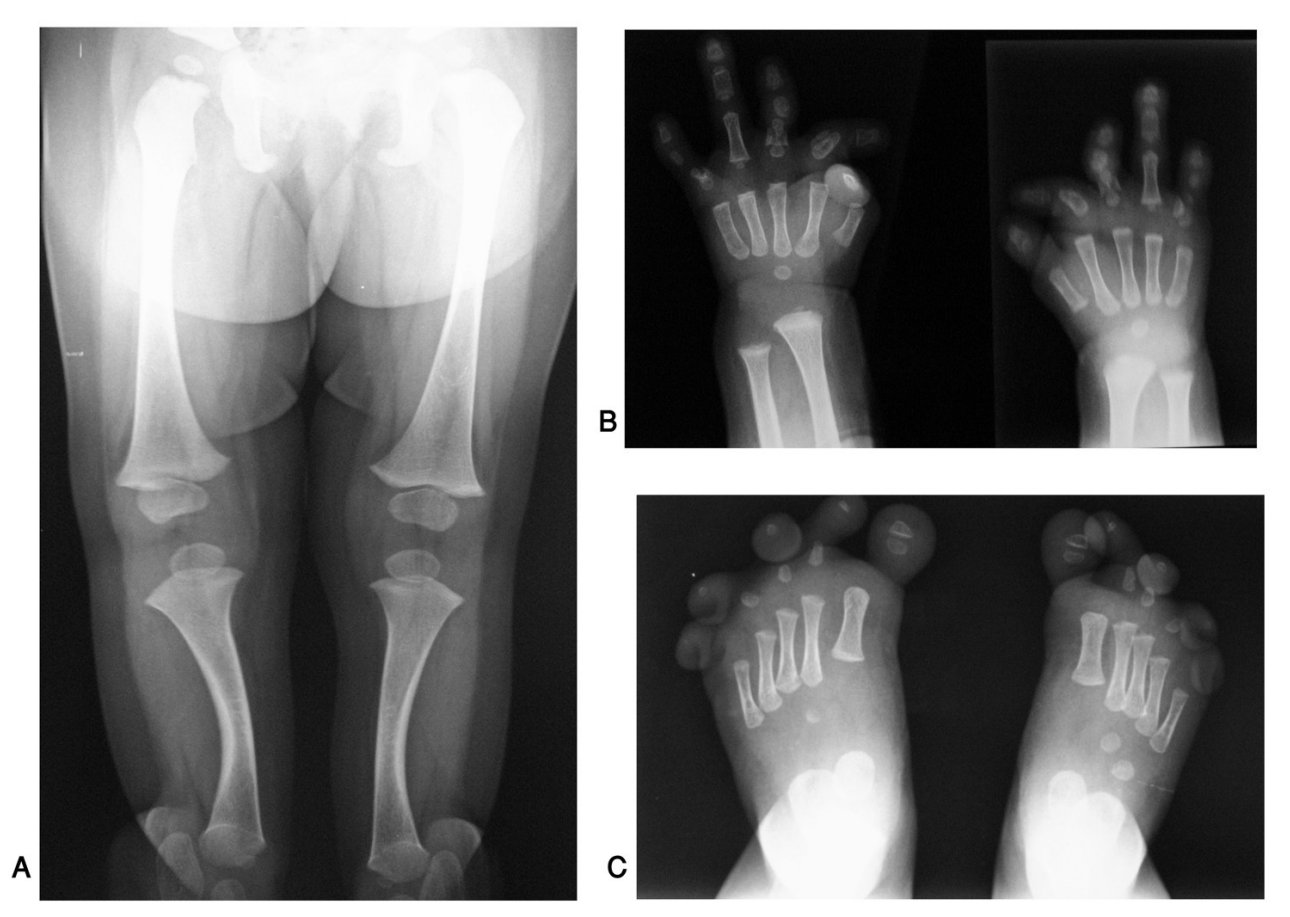
**An Egyptian patient with du Pan syndrome**. Note absence of both fibulae (A), X-ray of both hands and feet showing severe brachydactyly and radial deviation of fingers (B, C). (Limb Malformations & Skeletal Dysplasia Clinic, MSU, NRC).

**Figure 9:**
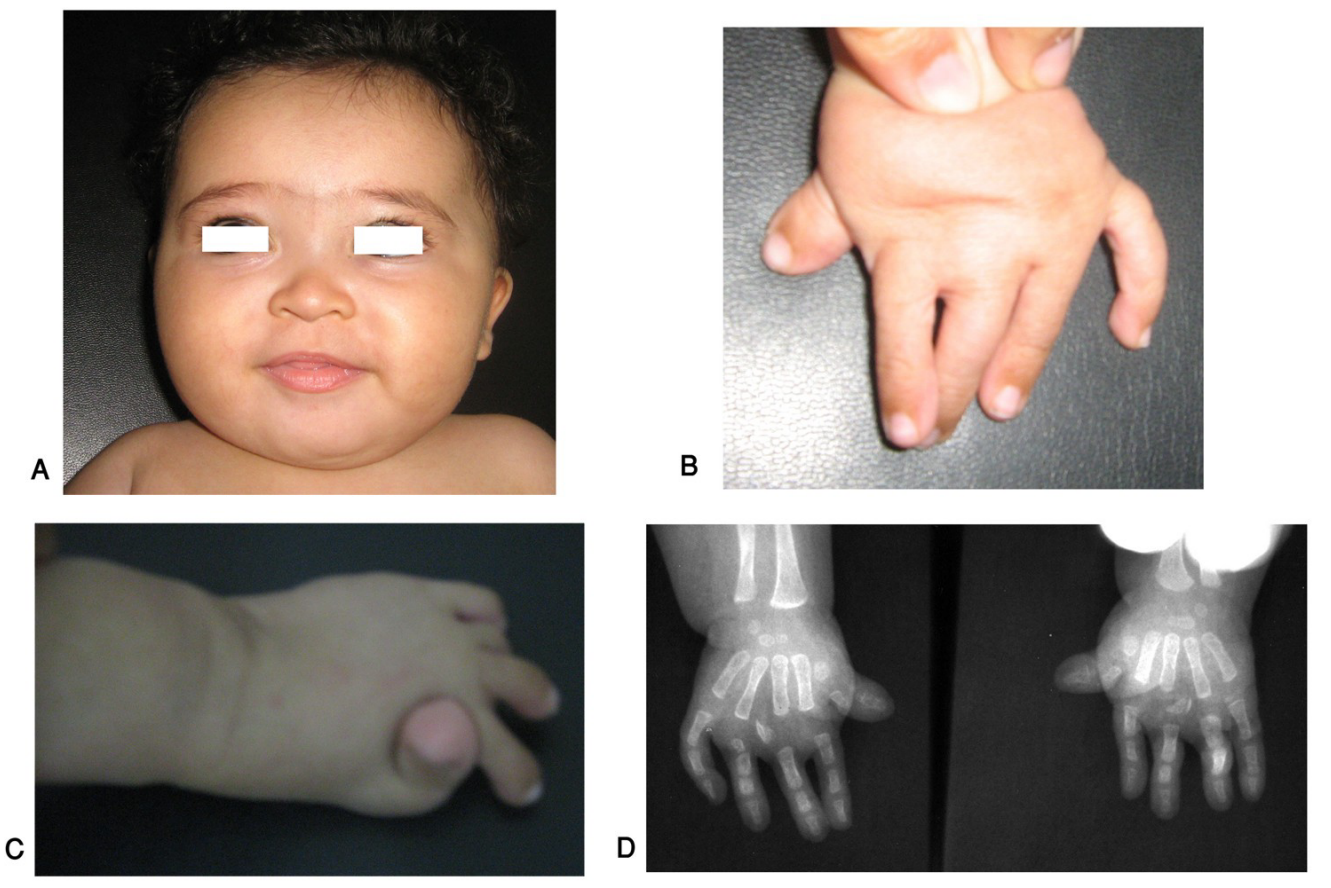
**An Egyptian patient with Temtamy preaxial brachydactyly syndrome**. Note round flat face (A), preaxial brachydactyly of hand and feet (B, C) and X-ray of both hands showing preaxial brachydactyly and hyperphalangism. (Limb Malformations & Skeletal Dysplasia Clinic, MSU, NRC).

The number of syndromes with brachydactyly is extensive. We have selected some of these syndromes in which we have special expertise and those that are most commonly included in their differential diagnosis. Additional file 1 shows different manifestations in the hands and in other parts of the body in the selected syndromes with brachydactyly (Figures 4, 5, 6, 7, 8 and 9). Table 3 shows the genomics of some syndromes with brachydactyly.

**Table 3:** Genomics of some syndromes with brachydactyly

**Type**	**OMIM**	**Gene Name**	**Gene Locus**	**Reference**
Robinow syndrome (autosomal recessive)	268310	*ROR2*	9q22	Stickler *et al*. [57]
Rubinstein-Taybi syndrome	180849	*CREBBP EP300*	16p13.3 22q13	Roelfsema & Peters [58]
Albright hereditary osteodystrophy	103580	*GNAS1 *(imprinting)	20q13.2-20q13.3	Davies & Hughes [59]
Brachydactyly type E with hypertension	112410	*PDE3A*	12p12.2-p11.2	Gong *et al*. [60]
du Pan syndrome	228900	*CDMP1*	20q11.2	Faiyaz-Ul-Haque *et al*. [61]

## Conclusion

The hand phenotype in isolated forms of brachydactyly allows identification of 11 types, with minimal degrees of phenotypic overlap. Typically, they show an autosomal dominant pattern of inheritance with variable expression and penetrance. The number of syndromic forms of brachydactyly is extensive, and, dictated by our expertise, only 21 entities were included in this review.

Molecular dysmorphology studies the abnormal function of molecules leading to disturbed development. Molecular analysis of isolated forms of brachydactyly has shed some light on the role of certain genes in normal human skeletogenesis and limb formation. Examples include *BMPR1B *gene mutations resulting in cartilage malformation and transmitted in a dominant negative manner. The *IHH *gene plays a pivotal role in human skeletogenesis. *HOXD13 *mutations result in a spectrum of limb disorders including brachydactyly. A modulating effect of the *ROR2 *gene on the BMP receptor pathway has been suggested. *CDMP1 *gene mutations with loss of function were found in certain types of isolated brachydactyly. It can be concluded that these genes affect the size and shape of individual bones or group of hand and foot bones. The mechanisms involve signaling molecules (*IHH); *conveying polarity information to cells (*ROR2*); and encoding the cartilage-derived morphogenetic protein (*DMPCI*). Further molecular studies are needed to unravel other underlying mechanisms of fine tuning of distal skeletal structures.

It is of utmost importance in cases of brachydactyly to proceed with clinical evaluation of both hands and feet including radiological evaluation, clinical evaluation of the whole body of the patient, family history (taking non-penetrance into account), and, if possible, clinical evaluation of at least first degrees relatives. If symptoms in other parts of the body are present, specific studies may be needed depending on the accompanying symptoms. Molecular analysis in isolated forms or syndromic cases of brachydactyly should be carried out if results could have consequences for patient care and/or for genetic counseling, or if indicated on research grounds.

## Competing interests

The authors declare that they have no competing interests.

## Authors' contributions

The two authors equally contributed to this review article.

## Consent

Written consent for publication of the clinical pictures was obtained from the parents of the patients.

## Supplementary Material

Additional file 1 Manifestations in some selected syndromes with associated brachydactyly. The data represent the digital phenotype, associated anomalies and mode of inheritance in some selected syndromes with brachydactyly. Click here for file

## References

[B1] Temtamy SA, McKusick VA (1978). The Genetics of Hand Malformations.

[B2] Superti-Furga A, Unger S (2007). Nosology and Classification of Genetic Skeletal Disorders: 2006 revision. Am J Med Genet A.

[B3] Hall CM (2002). International nosology and classification of constitutional disorders of bone. Am J Med Genet.

[B4] OMIM, Online Mendelian Inheritance in Man http://www.ncbi.nlm.nih.gov/Omim/searchomim.html.

[B5] Winter RM, Baraitser M (2006). The London Medical Database.

[B6] Bell J, Pensore LS (1951). On brachydactyly and symphalangism. Treasury of Human Inheritance.

[B7] Drinkwater H (1908). An account of a brachydactylous family. Proc Royal Soc Edin.

[B8] Drinkwater H (1912). Account of a family showing minor-brachydactyly. J Genet.

[B9] Drinkwater H (1914). A second brachydactylous family. J Genet.

[B10] Armour CM, Bulman DE, Hunter AG (2000). Clinical and radiological assessment of a family with mild brachydactyly type A1: the usefulness of metacarpophalangeal profiles. J Med Genet.

[B11] Farabee WC (1903). Hereditary and sexual influence in meristic variation: a study of digital malformations in man. PhD thesis.

[B12] Gao B, Guo J, She C, Shu A, Yang Z, Guo S, Feng G, He L (2001). Mutations in IHH, encoding Indian hedgehog, cause brachydactyly type A-1. Nat Genet.

[B13] McCready ME, Sweeney E, Fryer AE, Donnai D, Baig A, Racacho L, Warman ML, Hunter AG, Bulman DE (2002). A novel mutation in the IHH gene causes brachydactyly type A1: a 95-year-old mystery resolved. Hum Genet.

[B14] Kirkpatrick TJ, Au KS, Mastrobattista JM, McCready ME, Bulman DE, Northrup H (2003). Identification of a mutation in the Indian hedgehog (IHH) gene causing brachydactyly type A1 and evidence for a third locus [letter]. J Med Genet.

[B15] Mohr OL, Wriedt C (1919). A New Type of Hereditary Brachyphalangy in Man.

[B16] Jones GB (1964). Delta phalanx. J Bone Joint Surg.

[B17] Lehmann K, Seemann P, Stickler S, Sammar M, Meyer B, Suring K, Majewski F, Tinschert S, Grzescik KH, Muller D, Knaus P, Nurnberg P, Mundlos S (2003). Mutations in bone morphogenetic protein receptor 1B cause brachydactyly type A2. Proc Natl Acad Sci USA.

[B18] Kjaer KW, Eiberg H, Hansen L, Hagen CB van der, Rosendahl K, Tommerup N, Mundlos S (2006). A mutation in the receptor binding site of GDF5 causes Mohr-Wriedt brachydactyly type A2. J Med Genet.

[B19] Seemann P, Schwappacher R, Kjaer KW, Krakow D, Lehmann K, Dawson K, Stickler S, Pohl J, Ploger F, Staub E, Nickel J, Sebald W, Knaus P, Mundlos S (2005). Activating and deactivating mutations in the receptor interaction site of GDF5 cause symphalangism or brachydactyly type A2. J Clin Invest.

[B20] Sugiura Y (1962). Abnormalities of músculo-skeletal system observed in Shizuoka school children. J Hum Genet.

[B21] Hertzog K (1967). Shortened fifth medial phalanges. Am J Phys Anthropol.

[B22] Garn SM, Poznanski AK, Nagy JM, McCann MB (1972). Independence of brachymesophalangia-5 from brachymesophalangia-5 with cone mid-5. Am J Phys Anthropol.

[B23] Jeanselme (NI), Blamoutier (NI), Joannon (NI) (1923). Brachydactylie symetrique familiale: etude des lesions anatomique et de la transmission hereditaire. Rev Anthrop.

[B24] Stiles KA, Schalck J (1945). A pedigree of curved forefingers. J Hered.

[B25] Ohzeki T, Hanaki K, Motozumi H, Ohtahara H, Shiraki K, Yoshioka K (1993). Brachydactyly type A-4 (Temtamy type) with short stature in a Japanese girl and her mother. Am J Med Genet.

[B26] Zhao X, Sun M, Zhao J, Leyva JA, Zhu H, Yang W, Zeng X, Ao Y, Liu Q, Liu G, Lo WH, Jabs EW, Amzel LM, Shan X, Zhang X (2007). Mutations in HOXD13 underlie syndactyly type V and a novel brachydactyly-syndactyly syndrome. Am J Hum Genet.

[B27] Bass HN (1968). Familial absence of middle phalanges with nail dysplasia: a new syndrome. Pediatrics.

[B28] Cuevas-Sosa A, Garcia-Segur F (1971). Brachydactyly with absence of middle phalanges and hypoplastic nails: a new hereditary syndrome. J Bone Joint Surg Br.

[B29] MacKinder D (1857). Deficiency of fingers transmitted through six generations. Brit Med J.

[B30] Schwabe GC, Tinschert S, Buschow C, Meinecke P, Wolff G, Gillessen-Kaesbach G, Oldridge M, Wilkie AO, Komec R, Mundlos S (2000). Distinct mutations in the receptor tyrosine kinase gene ROR2 cause brachydactyly type B. Am J Hum Genet.

[B31] Lehmann K, Seemann P, Silan F, Goecke TO, Irgang S, Kjaer KW, Kjaergaard S, Mahonev MJ, Morlot S, Reissner C, Kerr B, Wilkie AO, Mundlos S (2007). A new subtype of brachydactyly type B caused by point mutations in the bone morphogenetic protein antagonist NOGGIN. Am J Hum Genet.

[B32] Galjaard RJ, Ham LI van der, Posch NA, Dijkstra PF, Oostra BA, Hovius SE, Timmenga EJ, Sonneveld GJ, Hoogeboom AJ, Heutink P (2001). Differences in complexity of isolated brachydactyly type C cannot be attributed to locus heterogeneity alone. Am J Med Genet.

[B33] Schwabe GC, Turkmen S, Leschik G, Palanduz S, Stover B, Goecke TO, Mundlos S (2004). Brachydactyly type C caused by a homozygous missense mutation in the prodomain of CDMP1. Am J Med Genet.

[B34] Castriota-Scanderbeg A, Garaci FG, Beluffi G (2005). Angel-shaped phalanges in brachydactyly C: a case report, and speculation on pathogenesis. Pediatr radiol.

[B35] Baraitser M, Burn J (1983). Recessively inherited brachydactyly type C. J Med Genet.

[B36] Debeer P, De Smet L, Fryns JP (2001). Intrafamilial clinical variability in type C brachydactyly. Genet Couns.

[B37] Polinkovsky A, Robin NH, Thomas JT, Irons M, Lynn A, Goodman FR, Reardon W, Kant SG, Brunner HG, Burgt I van der, Chitayat D, McGaughran J, Donnai D, Luyten FP, Warman ML (1997). Mutations in CDMP1 cause autosomal dominant brachydactyly type C [Letter]. Nature Genet.

[B38] Everman DB, Bartels CF, Yang Y, Yanamandra N, Goodman FR, Mendoza-Londono JR, Savarirayan R, White SM, Graham JM, Gale RP, Svarch E, Newman WG, Kleckers AR, Francomano CA, Govindaiah V, Singh L, Morrison S, Thomas JT, Warman ML (2002). The mutational spectrum of brachydactyly type C. Am J Med Genet.

[B39] Sammar M, Stickler S, Schwabe GC, Sieber C, Hartung A, Hanke M, Oishi I, Pohl J, Minami Y, Sebald W, Mundlos S, Knaus P (2004). Modulation of GDF5/BRI-b signaling through interaction with the tyrosine kinase receptor Ror2. Genes Cells.

[B40] Lehmann K, Seemann P, Boergermann J, Morin G, Reif S, Knaus P, Mundlos S (2006). A novel R486Q mutation in BMPR1B resulting in either a brachydactyly type C/symphalangism-like phenotype or brachydactyly type A2. Eur J Hum Genet.

[B41] Breitenbecher JK (1923). Hereditary shortness of thumbs. J Hered.

[B42] Poznanski AK, Werder EA, Giedion A (1977). The pattern of shortening of the bones of the hand in PHP and PPHP – a comparison with brachydactyly E, Turner syndrome, and acrodysostosis. Radiology.

[B43] Czeizel A, Göblyös P (1989). Familial combination of brachydactyly, type E and atrial septal defect, type II. Eur J Pediatr.

[B44] Hertzog KP (1968). Brachydactyly and pseudo-pseudohypoparathyroidism. Acta Genet Med Gemellol.

[B45] Hortling H, Puupponen E, Koski K (1960). Short metacarpal or metatarsal bones: pseudo-pseudohypoparathyroidism. J Clin Endocr.

[B46] McKusick VA, Milch RA (1964). The clinical behavior of genetic disease: selected aspects. Clin Orthop.

[B47] Pitt P, Williams I (1985). A new brachydactyly syndrome with similarities to Julia Bell types B and E. J Med Genet.

[B48] Jensen K, Hoo JJ (2004). Is brachydactyly type Ballard a variant of brachydactyly type E? [Letter]. Am J Med Genet.

[B49] Mollica F, Li Volti S, Guarneri B (1984). New syndrome: exostoses, anetodermia, brachydactyly. Am J Med Genet.

[B50] Johnson D, Kan SH, Oldridge M, Trembath RC, Roche P, Esnouf RM, Giele H, Wilkie AO (2003). Missense mutations in the homeodomain of HOXD13 are associated with brachydactyly types D and E. Am J Hum Genet.

[B51] Ray AK, Haldane JBS (1965). The genetics of a common Indian digital abnormality. Proc Nat Acad Sci.

[B52] Sugarman GI, Hager D, Kulik WJ (1974). A new syndrome of brachydactyly of the hands and feet with duplication of the first toes. Birth Defects Orig Art Ser.

[B53] Fujimoto A, Smolensky LS, Wilson MG (1982). Brachydactyly with major involvement of proximal phalanges. Clin Genet.

[B54] Kirner J (1927). Doppelseitige Verkrummungen des Kleinfingerendgliedes als selbstandiges Krankheitsbild. Fortschr Geb Roentgenstr Nuklearmed.

[B55] Saito TA (1963). Genetic study on the abnormal shortenings of the finger. Jap J Hum Genet.

[B56] David TJ, Burwood RL (1972). The nature and inheritance of Kirner's deformity. J Med Genet.

[B57] Stickler S, van Wijk Verhev, Witte F, Brieske N, Seidel K, Mundlos S (2006). Cloning and expression pattern of chicken Ror2 and functional characterization of truncating mutations in Brachydactyly type B and Robinow syndrome. Dev Dyn.

[B58] Roelfsema JH, Peters DJ (2007). Rubinstein-Taybi syndrome: clinical and molecular overview. Expert Rev Mol Med.

[B59] Davies SJ, Hughes HE (1993). Imprinting in Albright's hereditary osteodystrophy. J Med Genet.

[B60] Gong M, Zhang H, Schulz H, Lee A-A, Sun K, Bahring S, Luft FC, Nurnberg P, Reis A, Rohde K, Ganten D, Hui R, Hubner N (2003). Genome-wide linkage reveals a locus for human essential (primary) hypertension on chromosome 12p. Hum Molec Genet.

[B61] Faiyaz-Ul-Haque M, Ahmad W, Zaidi SH, Haque S, Teebi AS, Ahmad M, Cohen DH, Tsui LC (2002). Mutation in the cartilage-derived-morphogenetic protein-1 (CDMP1) gene in a kindred affected with fibular hypoplasia and complex brachydactyly (Du Pan syndrome). Clin Genet.

