# Supplementary material for: Brachydactyly
Source: Orphanet J Rare Dis. 2008 Jun 13;3:15. doi: 10.1186/1750-1172-3-15 (PMC2441618; doi:10.1186/1750-1172-3-15)
Supplement: Additional file 1 — Manifestations in some selected syndromes with associated brachydactyly. The data represent the digital phenotype, associated anomalies and mode of inheritance in some selected syndromes with brachydactyly. [file 1750-1172-3-15-S1.doc]

Additional file 1

**Manifestations in some selected syndromes with associated brachydactyly**

| **Syndrome & Synonyms** | **Digital phenotype** | **Associated anomalies** | **OMIM &**  **Inheritance** | **References** |
| --- | --- | --- | --- | --- |
| 1. **Brachydactyly-distal symphalangism**   **syndrome** | Resembles type A1 brachydactyly  with associated symphalangism.  Distal phalanx with the shape of a chess pawn | Scoliosis  Club foot  Tall stature | 113450,  Autosomal dominant (AD) | Sillence [1] |
| 1. **Thumbs, stiff with brachydactyly type A1 and developmental delay** | Resembles type A1 brachydactyly  With stiff thumbs | Mental retardation | 188201,  Dominant mutation  (DM) | Piussan *et al.* [2]  Barber *et al.* [3] |
| 1. **Brachydactyly type A2 with microcephaly** | Resembles type A2 brachydactyly | Microcephaly  Seizure disorder with  generalized spike-wave  discharges by EEG  Diabetes mellitus | 211369,  Autosomal recessive (AR) | Graham [4] |
| 1. **Microcephaly and digital anomalies with normal intelligence** | Brachydactyly with radial  clinodactyly of 4^th^ & 5^th^ digits and  ulnar clinodactyly of 2^nd^ digits. Increased space between 2^nd^ & 3^rd^ digits, an abnormal palmar crease (Sydney line), short toes with syndactyly between 4^th^ & 5^th^ toes | Microcephaly  Chronic nephritis | 602585, AD | Kawame *et al.* [5]  Teszas *et al.* [6] |
| 1. **Brachydactyly type A 6**  (BDA6) (*BRACHYMESOPHALANGY WITH MESOMELIC SHORT LIMBS AND CARPAL AND TARSAL OSSEOUS ABNORMALITIES, OSEBOLD-REMONDINI SYNDROME*) | Hypoplastic or absent middle  phalanges of hands & feet  Radial deviation of index fingers | Short stature  Mesomelic dwarfism  Delayed coalescence of  bipartite calcanei  (in younger members).  Hamate and capitate fusion  Normal intelligence | 112910, AD | Osebold *et al.* [7] |
| 1. **Robinow syndrome** | Type D brachydactyly with short metacarpals (Figs. 4C,D & 5A) | Short stature  Mesomelic dwarfism (Fig. 5A)  Supernumerary teeth  Umbilical hernia  Characteristic fetal face  (Fig. 4A)  Hypogenitalism (Fig. 4B)  Gingival hypertrophy  (Fig. 5B)  Multiple ribs, vertebral anomalies, hemivertebrae & scoliosis more in common in AR type  (Fig. 5C) | 180700, AD  268310, AR | Temtamy *et al.* [8]  Mazzeu *et al.* [9]  Meguid & Aglan [10]  Stickler *et al.* [11]  Ali *et al.* [12] |
| 1. **Coloboma of macula with**   **type B brachydactyly**  (syn: *APICAL DYSTROPHY, SORSBY SYNDROME*) | Type B brachydactyly | Bilateral pigmented macular coloboma  Absent kidney (unilateral or bilateral)  Sensorineural hearing loss | 120400, AD | Sorsby [13]  Thompson &  Baraitser [14]  Bacchelli *et al.* [15] |
| 1. **Temtamy syndrome of**   **craniofacial dysmorphism,**  **ocular coloboma,**  **absent corpus callosum & aortic dilatation** | Brachydactyly of hands and feet  mainly of metacarpals 3-5 | Mental retardation  Club feet  Agenesis of corpus callosum  Cerebral ventricular  enlargement  Eye colobomas  Genu vara  Aortic dilatation  Craniofacial dysmorphism | 218340, AR | Temtamy *et al.* [16]  Chan *et al.* [17]  Ramocki *et al.* [18]  Talisetti *et al.* [19]  Li et al. [20] |
| 1. **Brachydactyly,**   **coloboma & anterior**  **segment dysgenesis** | Short digits and clinodactyly | Anterior segment dysgenesis  Ocular coloboma  Mild learning disability  Mild hearing impairment  Cleft palate  Short stature | 610023, AD,  X-linked can not be  excluded | Quinn *et al.* [21] |
| 1. **Hirschsprung disease**   **with type D brachydactyly** | Absence or short distal phalanx &  nails of thumbs & great toes | Hirschsprung disease | 306980, X-linked | Reynolds *et al.* [22] |
| 1. **Rubinstein-Taybi**   **Syndrome** (syn: *RSTS, BROAD-THUMB HALLUX SYNDROME*) | Broad thumbs & big toes  (Fig. 6B, C, D) | Characteristic facies with long nose & antimongoloid slanting  of eyes (Fig. 6A)  Cardiac anomalies  Dental anomalies  Slipped capital femoral  epiphysis  Mental retardation | 180849, DM | Rubinstein and Taybi  [23]  Bloch-Zupan *et al.*  [24]  Roelfsema & Peters  [25] |
| 1. **Albright hereditary osteodystrophy**   (syn: *AHO,PSEUDOHYPOPARATHYROIDISM, PHP, pseudo-pseudohypoparathyroidism,PPHP*) | Type E brachydactyly  (Typically, shortening of III, IV,  V metacarpals and I distal  phalanx) | Resistance to multiple  hormones that work via  cAMP  Short stature  Obesity & round facies  Subcutaneous ossifications  Pseudohypoparathyroidism  Hypocalcemia & elevated  PTH levels  Patients with PPHP have  normal Ca & PTH levels | 103580, AD | Davies & Hughes [26]  Wilson *et al.* [27]  Mouallem *et al.* [28] |
| 1. **Brachydactyly-Mental Retardation syndrome** (syn: *BDMR - ALBRIGHT HEREDITARY OSTEODYSTROPHY-LIKE SYNDROME - ALBRIGHT HEREDITARY OSTEODYSTROPHY 3; AHO3*) | Type E brachydactyly | Short stature  Stocky build  Mental retardation  Eczema  Normal PTH and Ca  metabolism  Normal levels of Gs-alpha  and de novo deletions  of 2q37 | 600430, sporadic | Wilson *et al.* [29]  Shrimpton *et al.* [30] |
| Brachydactyly type E with hypertension (syn: *HTNB, BRACHYDACTYLY WITH HYPERTENSION, BRACHYDACTYLY, TYPE E, WITH SHORT STATURE AND HYPERTENSION, BILGINTURAN SYNDROME*) | Type E brachydactyly | Essential hypertension,  Diminished baroreflex  sensitivity with markedly  improved blood pressure  buffering,  Loops in the posterior/  inferior cerebellar artery by  MRI angiography of posterior fossa,  No significant  ophthalmologic findings of prolonged hypertension,  Hypertension is medically responsive. | 112410, AD | Bilginturan *et al.* [31]  Luft *et al.* [32]  Schuster *et al.* [33]  Bahring *et al.* [34]  Nagai *et al.* [35]  Bahring *et al.* [36]  Chitayat *et al.* [37]  Toka *et al.* [38]  Hattenbach *et al.* [39]  Schuster *et al.* [40]  Gong *et al.* [41] |
| 1. **Brachydactyly,**   **long-thumb type** | Symmetric brachydactyly and  relatively long thumbs. Tip of  thumb extended distal to the  proximal interphalangeal joint of  index finger when digits are  opposed. | Skeletal & joint anomalies  Cardiac conduction defects | 112430, AD | [Hollister and Hollister](javascript:Anchor('112430_Reference1'))  [42] |
| 1. **Digital arthropathy-brachydactyly, familial** (syn: *FDAB*) | Progressive brachydactyly of  middle & distal phalanges of hands & feet with onset in the first decade  of life. | Progressive arthropathy of the interphalangeal, metacarpophalangeal & metatarsophalangeal joints  Proximal articular surfaces  become flattened & deformed  Changes are more marked in  hands than feet  Presumably subchondral  pathology primarily affects  heads of phalanges,  metacarpals & metatarsals | 606835, AD | Amor *et al.* [43] |
| 1. **Ulnar/fibular ray defect and brachydactyly** | Brachydactyly of digits | Ulnar/fibular hypoplasia  Ulnar ray defects  Midface hypoplasia  Atrial septal defects & hemangioma  Normal mammary tissue &  Sweating  Short stature | 608571, AD | Morava *et al.* [44] |
| 1. **Fibular hypoplasia and complex brachydactyly** (syn: *du Pan SYNDROME,*   *GREBE SYNDROME, MESOMELIA-TYPE HUNTER-THOMPSON*) | Complex type of brachydactyly:  Short various metacarpals, small carpals, trapezoid middle phalanx  of index finger with radial deviation  Short laterally deviated  hypoplastic toes (ball-like)  (Figs. 7 & 8) | Bilateral absence of fibula  (Fig. 8A)  Tibiotarsal dislocation  (Volkmann deformity)  Short limbs | 228900, AR | Martin du Pan [45]  Grebe [46]  Temtamy &  McKusick [47]  Kohn *et al.* [48]  Szczaluba *et al.* [49]  Ahmad *et al.* [50]  Faiyaz-Ul-Haque *et al.* [51] |
| 1. **Brachydactyly- Mononen type** (syn: *THUMBS AND GREAT TOES, SHORT AND ABDUCTED*) | Preaxial brachydactyly & abducted thumbs & big toes  X-ray hands & feet show short 1^st^ metacarpals & 1^st^ metatarsals &  absent distal phalanges of index fingers & 2^nd^ toes  Coalescence of carpal & tarsal bones | Mild short stature  Bow legs with proximal overgrowth of fibula | 301940,  X-linked dominant  (XLD) | Mononen *et al.* [52] |
| Brachydactyly, preaxial, with hallux varus and thumb abduction **(syn:** *CHRISTIAN BRACHYDACTYLY*) | Short abducted thumbs & big toes  Short metacarpals, metatarsals &  distal phalanges | Mental retardation | 112450, AD | Christian *et al.* [53] |
| Temtamy preaxial brachydactyly syndrome (syn: *PREAXIAL BRACHYDACTYLY SYNDROME, TEMTAMY TYPE*) | Bilateral symmetrical preaxial brachydactyly and  Hyperphalangism of digits 1-3  Ulnar deviation of 5^th^ fingers & accessory ossicles of digits 2-5 & abnormal phalanges of thumbs  (Fig. 9) | Mental retardation  Growth retardation  Sensorineural deafness  Rounded facies (Fig, 9A)  Micrognathia  Talon cusps of upper central incisors  Cleft palate | 605282, AR | Temtamy *et al.* [54]  Clarkson *et al.* [55]  Temtamy [56]  Temtamy *et al.* [unpublished  observations] |

**References**

1. Sillence DO: **Brachydactyly, distal symphalangism, scoliosis, tall stature, and club feet: a new syndrome.** *J Med Genet* 1978, **15:** 208-211.

2. Piussan C, Lenaerts C, Mathieu M, Boudailliez B: **Dominance reguliere d'une ankylose des pouces avec retard mental se transmettant sur trois generations.** *J Genet Hum* 1983, **31:** 107-114.

3. Barber ND, Carpenter NJ, Say B: **Bilateral ankylosed thumbs and mental retardation [Letter].** *Am J Med Genet* 1990, **36:** 367.

4. Graham JMJr: **New syndrome of type A2 brachydactyly, microcephaly, and diabetes in siblings born to consanguineous parents [Abstract].** *Am J Hum Genet* 1989, **Suppl 45:** A76.

5. Kawame H, Pagon RA, Hudgins L: **Digital anomalies, microcephaly, and normal intelligence: new syndrome or Feingold syndrome?** *Am J Med Genet* 1997, **69:** 240-244.

6. Teszas A, Meijer R, Scheffer H, Gyuris P, Kosztolanyi G, van Bokhoven H, Kellermayer R: **Expanding the clinical spectrum of MYCN-related Feingold syndrome.** *Am J Med Genet* 2006, **140A:** 2254-2256

7. Osebold WR, Remondini DJ, Lester EL, Spranger JW, Opitz JM: **An autosomal dominant syndrome of short stature with mesomelic shortness of limbs, abnormal carpal and tarsal bones, hypoplastic middle phalanges, and bipartite calcanei.** *Am J Med Genet* 1985, **22:** 791-809.

8. Temtamy SA, El-Badry TH, Aboul-Ezz EHA: **Clinical, orodental and electronmicroscopic changes of gingival biopsy in autosomal recessive Robinow syndrome suggest a storage disorder and a midline developmental field defect.** *Egypt Med J NRC* 2004, **5:** 149-163.

9. Mazzeu JE, Pardono E, Vianna-Morgante AM, Richieri-Costa A, Ae Kim C, Brunoni D, Martelli L, de Andrade CE, Colin G, Otto PA: **Clinical characterization of autosomal dominant and recessive variants of Robinow syndrome.** *Am J Med Genet* 2007, **143:** 320-325.

10. Meguid NA, Aglan MS: Clinical and anthropometric study in Egyptian children with Robinow Syndrome. *The Gaz Egypt Paed* 2002, **50:** 399-413.

11. Stickler S, Verhev van Wijk, Witte F, Brieske N, Seidel K, Mundlos S: **Cloning and expression pattern of chicken Ror2 and functional characterization of truncating mutations in Brachydactyly type B and Robinow syndrome.** *Dev Dyn* 2006, **235:** 3456-3465.

12. Ali BR, Jeffery S, Patel N, Tinworth LE, Meguid N, Patton MA, Afzal AR: **Novel Robinow syndrome causing mutations in the proximal region of the frizzled-like domain of ROR2 are retained in the endoplasmic reticulum.** *Hum Genet* 2007, **122:** 389-395.

13. Sorsby A: **Congenital coloboma of the macula, together with an account of the familial occurrence of bilateral macular coloboma in association with apical dystrophy of hands and feet.** *Brit J Ophthal* 1935, **19:** 65-90.

14. Thompson EM, Baraitser M: **Sorsby syndrome: a report on further generations of the original family.** *J Med Genet* 1988, **25:** 313-321.

15. Bacchelli C, Wilson LC, Cook JA, Winter RM, Goodman FR: **ROR2 is mutated in hereditary brachydactyly with nail dysplasia, but not in Sorsby syndrome [Letter].** *Clin Genet* 2003, **64:** 263-265.

16. Temtamy SA, Salam MA, Aboul-Ezz EH, Hussein HA, Helmy SA, Shalash BA: **New autosomal recessive multiple congenital abnormalities/mental retardation syndrome with craniofacial dysmorphism absent corpuss callosum, iris colobomas and connective tissue dysplasia.** *Clin Dysmorphol* 1996, **5:** 231-240.

17. Chan AK, Levin AV, Teebi AS: **Craniofacial dysmorphism, agenesis of corpus callosum and ocular colobomas: Temtamy syndrome?** *Clin Dysmorphol* 2000, **9:** 223-226.

18. Ramocki MB, Dowling J, grinberg I, Kimonis VE, Cardoso C, Gross A, Chung J, Martin CL, Ledbetter DH, Dobyns WB, Millen KJ: **Reciprocal fusion transcripts of two novel Zn-finger genes in a female with absence of corpuss callosum, ocular colobomas and a balanced translocation between chromosomes 2p24 and 9q32.** *Eur J Hum Genet* 2003, **11:** 527-534.

19. Talisetti A, Forrester SR, Gregory D, Johnson L, Schneider MC, Kimonis VE: **Temtamy-like syndrome associated with translocation of 2p24 and 9q32.** *Clin Dysmorphol* 2003, **12:** 175-177.

20. Li J, Shivakumar S, Wakahiro M, Mukherjee P, Barkovich AJ, Slavotinek A, Sherr EH: **Agenesis of corpus callosum, optic coloboma, intractable seizures, craniofacial and skeletal dysmorphisms: An autosomal recessive disorder similar to Temtamy syndrome.** *Am J Med Genet Part A* 2007, **143A:** 1900-1905.

21. Quinn SM, Black GC, Biswas S, Clayton-Smith J, Lloyd IC: **Autosomal dominant brachydactyly, coloboma and anterior segment dysgenesis.** *Ophthalmic Genet* 2004, **25:** 277-283.

22. Reynolds JF, Barber JC, Alford BA, Chandler JG, Kelly TE: **Familial Hirschsprung disease and type D brachydactyly: a report of four affected males in two generations.** *Pediatrics* 1983, 71: 246-249.

23. Rubinstein JH, Taybi H: **Broad thumbs and toes and facial abnormalities.** *Am J Dis Child* 1963, **105:** 588-608.

24. Bloch-Zupan A, Stachtou J, Emmanouil D, Arveiler B, Griffiths D, Lacombe D: **Oro-dental features as useful diagnostic tool in Rubinstein-Taybi syndrome.** *Am J Med Genet* 2007, **143A:** 570-573.

25. Roelfsema JH, Peters DJ: **Rubinstein-Taybi syndrome: clinical and molecular overview.** *Expert Rev Mol Med* 2007, **20:** 1-16.

26. Davies SJ, Hughes HE: **Imprinting in Albright's hereditary osteodystrophy.** *J Med Genet* 1993, **30:** 101-103.

27. Wilson LC, Oude Luttikhuis MEM, Clayton PT, Fraser WD, Trembath RC: **Parental origin of Gs-alpha gene mutations in Albright's hereditary osteodystrophy.** *J Med Genet* 1994, **31:** 835-839.

28. Mouallem M, Shaharabany M, Weintrob N, Shalitin S, Nagelberg N, Shapira H, Zadik Z, Farfel Z: **Cognitive impairment is prevalent in pseudohypoparathyroidism type Ia, but not in pseudo pseudohypoparathyroidism: possible cerebral imprinting of Gsalpha [Abstract].** *Clin Endocrinol* 2007.

29. Wilson LC, Leverton K, Oude Luttikhuis MEM, Oley CA, Flint J, Wolstenholme J, Duckett DP, Barrow MA, Leonard JV, Read AP, Trembath RC: **Brachydactyly and mental retardation: an Albright hereditary osteodystrophy-like syndrome localized to 2q37.** *Am J Hum Genet* 1995, **56:** 400-407.

30. Shrimpton AE, Braddock BR, Thomson LL, Stein CK, Hoo JJ: **Molecular delineation of deletions on 2q37.3 in three cases with an Albright hereditary osteodystrophy-like phenotype.** *Clin Genet* 2004, **66:** 537-544.

31. Bilginturan N, Zileli S, Karacadag S, Pirnar T: **Hereditary brachydactyly associated with hypertension.** *J Med Genet* 1973, **10:** 253-259.

32. Luft FC, Toka O, Toka HR, Jordan J, Bahring S: **Mendelian hypertension with brachydactyly as a molecular genetic lesson in regulatory physiology.** *Am J Physiol Regul Integr Comp Physiol* 2003, **285:** R709-714.

33. Schuster H, Wienker TF, Toka HR, Bahring S, Jeschke E, Toka O, Busjahn A, Hempel A, Tahlhammer C, Oelkers W, Kunze J, Bilginturan N, Haller H, Luft FC: **Autosomal dominant hypertension and brachydactyly in a Turkish kindred resembles essential hypertension.** *Hypertension* 1996, **28:** 1085-1092.

34. Bahring S, Schuster H, Wienker TF, Haller H, Toka H, Toka O, Naraghi R, Luft FC: **Construction of a physical map and additional phenotyping in autosomal-dominant hypertension and brachydactyly, which maps to chromosome 12 [Abstract].** *Am J Hum Genet* 1996, **Suppl 59**: A55.

35. Nagai T, Nishimura G, Kato R, Hasegawa T, Ohashi H, Fukushima Y: **Del(12)(p11.21p12.2) associated with an asphyxiating thoracic dystrophy or chondroectodermal dysplasia-like syndrome.** *Am J Med Genet* 1995, **55:** 16-18.

36. Bahring S, Nagai T, Toka HR, Nitz I, Toka O, Aydin A, Muhl A, Wienker TF, Schuster H, Luft FC: **Deletion at 12p in a Japanese child with brachydactyly overlaps the assigned locus of brachydactyly with hypertension in a Turkish family [Letter].** *Am J Hum Genet* 1997, **60:** 732-735.

37. Chitayat D, Grix A, Balfe JW, Abramowicz JS, Garza J, Fong CT, Silver MM, Saller, DNJr, Bresnick GH, Giedion A, Lachman RS, Rimoin DL: **Brachydactyly-short stature-hypertension (Bilginturan) syndrome: report on two families.** *Am J Med Genet* 1997, **8:**285.

38. Toka HR, Bahring S, Chitayat D, Melby JC, Whitehead R, Jeschke E, Wienker TF, Toka O, Schuster H, Luft FC: **Families with autosomal dominant brachydactyly type E, short stature, and severe hypertension.** *Ann Intern Med* 1998*,* **129:** 204-208.

39. Hattenbach L.-O, Toka HR, Toka O, Schuster H, Luft FC: **Absence of hypertensive retinopathy in a Turkish kindred with autosomal dominant hypertension and brachydactyly.** *Brit J Ophthal* 1998, **82:** 1363-1365.

40. Schuster H, Wienker TF, Bahring S, Bilginturan N, Toka HR, Neitzel H, Jeschke E, Toka O, Gilbert D, Lowe A, Ott J, Haller H, Luft FC: **Severe autosomal dominant hypertension and brachydactyly in a unique Turkish kindred maps to human chromosome 12.** *Nature Genet* 1996, **13:** 98-100.

41. Gong M, Zhang H, Schulz H, Lee A-A, Sun K, Bahring S, Luft FC, Nurnberg P, Reis A, Rohde K, Ganten D, Hui R, Hubner N: **Genome-wide linkage reveals a locus for human essential (primary) hypertension on chromosome 12p.** *Hum Molec Genet* 2003, **12:** 1273-1277.

41. Hollister DW, Hollister WG: **The 'long-thumb' brachydactyly syndrome.** *Am J Med Genet* 1981, **8:** 5-16.

43. Amor DJ, Tudball C, Gardner RJ, Lamande SR, Bateman JF, Savarirayan R: **Familial digital arthropathy-brachydactyly.**  *Am J Med Genet* 2002, **108:** 235-240.

44. Morava E, Czako M, Karteszi J, Cser B, Weissbecker K, Mehes K: **Ulnar/fibular ray defect and brachydactyly in a family: a possible new autosomal dominant syndrome.** *Clin Dysmorphol* 2003, **12:** 161-165.

45. Martin du Pan C: **Absence congenitale du perone sans deformation du tibia: curieuses deformations congenitales des mains.** *Rev Orthop* 1924, **11:** 227-234.

46. Grebe H: **Chondrodysplasie***.* Rome: Istituto Gregorio Mendel; 1955: 300-303.

47. Temtamy SA, McKusick VA: *The Genetics of Hand Malformations.* New York: Alan R Liss, INC.; 1978.

48. Kohn G, Veder M, Schoenfeld A, El Shawwa R: **New type of autosomal recessive short-limb dwarfism with absent fibulae, exceptionally short digits, and normal intelligence.** *Am J Med Genet* 1989, **34:** 535-540.

49. Szczaluba K, Hilbert K, Obersztyn E, Zabel B, Mazurczak T, Kozlowski K: **Du Pan syndrome phenotype caused by heterozygous pathogenic mutations in CDMP1 gene.** *Am J Med Genet* 2005, **138A:** 379-383.

50. Ahmad M, Abbas H, Wahab A, Haque S: **Fibular hypoplasia and complex brachydactyly (Du Pan syndrome) in an inbred Pakistani kindred.** *Am J Med Genet* 1990, **36:** 292-296.

51. Faiyaz-Ul-Haque M, Ahmad W, Zaidi SH, Haque S, Teebi AS, Ahmad M, Cohen DH, Tsui LC: **Mutation in the cartilage-derived-morphogenetic protein-1 (CDMP1) gene in a kindred affected with fibular hypoplasia and complex brachydactyly (Du Pan syndrome).** *Clin Genet* 2002, **61:** 454-458.

52. Mononen TK, Karnes PS, Senac MOJr, Falk RE: **New skeletal dysplasia with unique brachydactyly.** *Am J Med Genet* 1992*,* **42:** 706-713.

53. Christian JC, Cho KS, Franken EA, Thompson BH: **Dominant preaxial brachydactyly with hallux varus and thumb abduction.** *Am J Hum Genet* 1972, **24:** 694-701.

54. Temtamy SA, Meguid NA, Ismail SI, Ramzy MI: **A new multiple congenital anomaly, mental retardation syndrome with preaxial brachydactyly, hyperphalangism, deafness and orodental anomalies.** *Clin Dysmorphol* 1998, **7:** 249-255.

55. Clarkson JHW, Homfray T, Heron CW, Moss AL: **Catel-Manzke syndrome: A report of a female with severely malformed hands and feet. An extension of the phenotype or a new syndrome?** *Clin Dysmorphol* 2004, **13:** 237-240.

56. Temtamy SA: **Catel-Manzke digitopalatal syndrome or Temtamy preaxial brachydactyly hyperphalangism syndrome?** *Clin Dysmorphol* 2005, **14:**211.
